# Sudden cardiac arrest in a child with nemaline myopathy

**DOI:** 10.1186/s13052-015-0124-8

**Published:** 2015-03-21

**Authors:** Lucia Marseglia, Gabriella D’Angelo, Sara Manti, Vincenzo Salpietro, Teresa Arrigo, Vittorio Cavallari, Eloisa Gitto

**Affiliations:** Neonatal and Pediatric Intensive Care Unit, Department of Pediatrics, University of Messina, Italy, Via Consolare Valeria 1, 98125 Messina, Italy; Unit of Paediatric Genetics and Immunology, Department of Paediatrics, University of Messina, Messina, Italy; Ultrastructural Pathology Unit, University of Messina, Messina, Italy

**Keywords:** Nemaline myopathy, Cardiac arrest, Hypoxic-ischemic brain injury, Child

## Abstract

**Background:**

Nemaline myopathy is a rare, non progressive congenital skeletal muscle disorder defined by the presence of inclusions known as nemaline rods in muscle fibers. Several clinical subtypes have been described, according to degree of muscle weakness, severity and age at onset. The course of nemaline myopathy is very slowly progressive, and death is usually due to respiratory failure. Cardiac involvement is rare and generally considered to be the result of ACTA1 mutations.

**Patient:**

We report the case of a 6 year old boy with typical congenital nemaline myopathy. Nemaline myopathy was confirmed at 3 years of age by muscle biopsy. No mutation of ACTA1, TPM2 and TNNT1 genes was detected. The child died suddenly of cardiac arrest and associated hypoxic-ischemic brain injury, in absence of acute respiratory failure or swallowing difficulties.

**Results:**

Nemaline cardiomyopathy was suspected, but post mortem cardiac biopsy did not show findings consistent with nemaline myopathy.

**Conclusions:**

Congenital typical nemaline myopathy is not necessarily a static or very slowly progressive disorder and acute cardiac deterioration can lead to early death.

## Background

Nemaline myopathy (NM) was first described as a non progressive congenital skeletal muscle disorder defined by the presence of inclusions known as nemaline rods in muscle fibers [1]. Its estimated incidence is of 1:50,000 live births [2]. According to the degree of muscle weakness, severity, and age at onset, 6 clinical subtypes of NM have been described by the European Neuromuscular Centre International Workshop: severe (neonatal) congenital (16%), Amish (1%), intermediate congenital (20%), typical congenital (46%), childhood-onset (13%), and adulthood-onset (4%) forms [3]. Clinical subtypes reflect the clinical heterogeneity of this disease. The most common classical form is characterized by onset in early infancy or childhood with hypotonia or general weakness predominantly affecting facial, axial, and proximal limb muscles. Additional clinical features include skeletal deformities, dysmorphic face, high-arched palate, and respiratory distress associated with respiratory infections [4].

In making the diagnosis of NM, clinical suspicion is the first step. Creatine kinase may be normal or slightly elevated. Electromyography may show myopathic changes such as action potentials of small amplitude and short duration [4], but these changes are not specific. Diagnosis is confirmed with muscle biopsy, which shows characteristic rod bodies, best seen with modified Gomori trichrome staining. Rods are typically found in the sarcoplasm but may also be intranuclear [4]. Rod formation has been associated with mutations in skeletal muscle α-actin (ACTA1) [5], nebulin (NEB) [6], tropomyosin 3 (TPM3) [7], tropomyosin 2 (TPM2) [8], troponin T1 (TNNT1) [9], cofilin (CLF2) [10], and, most recently, a member of the BTB/Kelch family of proteins (KBTBD13) [11].

Respiratory failure, constant in more severe types, can occur also in patients with mild presentation. Diaphragmatic weakness is a prominent cause of respiratory failure. Unilateral hemidiaphragm weakness is relatively common. Bilateral diaphragmatic weakness remains rare, and the cause is often not established [12].

Cases with cardiac involvement have rarely been described [13].

We describe here the case of a 6 year old child with nemaline myopathy who died of sudden cardiac arrest in absence of respiratory failure.

### Case report

A 6 year-old Romanian male was born full-term via spontaneous vaginal delivery. There was no known maternal exposure to teratogens or infections. He was the first child of healthy, unrelated parents, with no family history of neuromuscular disorders. Apgar score at 1 and 5 min was 8 and 9 respectively. Systemic physical examination revealed elongated facies, arched palate, external feet rotation, abducted hands, abducted thumbs, distal arthrogryposis. Cerebral ultrasound scan revealed essentially normal findings. Mild hypotonia was detected in the neonatal period. The child was able to hold his head steady and upright by 6 months of age. At 8 months, he was sitting with self-propping (hands pushing down on legs while sitting). He began walking with support at 30 months of age, and could achieve a standing position using a Gowers’ manoeuvre. There was moderate and symmetrical weakness in abduction and adduction movements, with the prominent involvement of distal and axial muscles. Motor coordination was normal and tendon reflexes deep were depressed. At 3 years of age, he presented a high-stepping gait with bilateral foot drop, moderate joint contractures, scoliosis and kyphosis convex to the left.

At 3 years of age, the nemaline myopathy was confirmed by muscle biopsy. Patient karyotype was 46, XY. No mutation of ACTA1 gene, TPM2 gene and TNNT1 gene was detected. According to clinical presentation, the typical congenital form of nemaline myopathy was diagnosed.

During follow up, he showed normal cardiovascular and respiratory functions. Pulmonary function tests were unremarkable, nocturnal oximetry test, electrocardiography, and echocardiography were also normal. Magnetic resonance imaging of the brain did not detect abnormalities. He could walk, sit and use upper extremities for eating and writing. Cognitive and speech development were normal. Despite the myopathy, he had no relevant problems in daily life.

At 6 years of age, coming back from school by car, he reported a sudden cardiac arrest. Rescuers performed cardiopulmonary resuscitation (RCP), and, placing the defibrillator probes, documented the asystole. No food or fluid were detected in the upper airways. After 20 min of RCP, return of spontaneous circulation was achieved, and the child, intubated and mechanically ventilated, was admitted to the Pediatric intensive care unit in coma.

Chest X-ray was unremarkable. Echocardiography showed multiple intracardiac thrombi, as is typically found after a prolonged cardiac arrest, with an ejection fraction of 69%.

EGC showed a sinus rhythm with normal atria-ventricular conduction.

**Figure 1:**
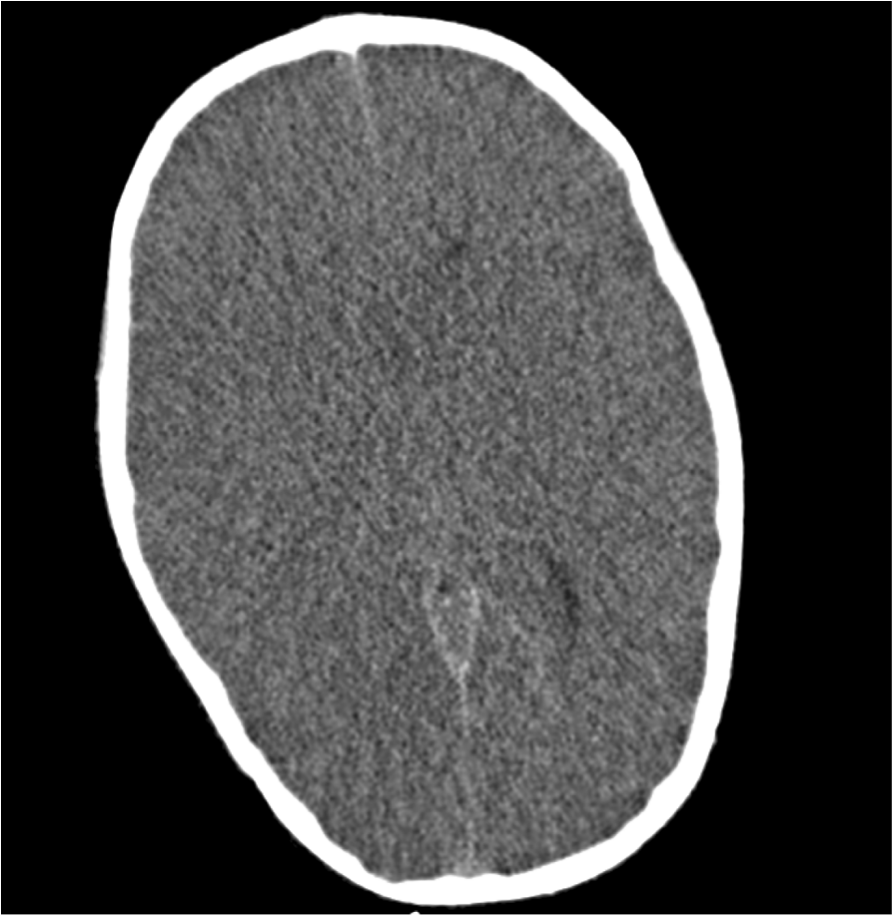
**CT scan of the brain.** Loss of cortical convolutions; reduction of cisternal cerebral spaces; reduced gray to white matter tissue intensity contrast.

Computed tomography (CT) scan of the brain demonstrated loss of cortical convolutions; reduction of cisternal cerebral spaces; non visualization of the entire cavity of the third ventricle, of the temporal horn, of the fourth ventricle; reduced grey to white matter tissue intensity contrast [Figure 1].

**Figure 2:**
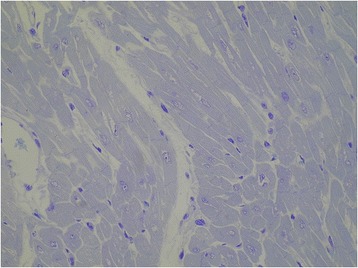
**Electron microscopy of the cardiac muscle.** Resin section, toluidine blue. Original magnification 400 X. Myocardiocytes showed preserved ultrastructure, with myofibrils regularly arranged and no changes in endoplasmic reticulum and mitochondria.

The patient succumbed to hypoxic-ischemic brain injury caused by cardiac arrest 7 days after admission. Corneas, liver and kidneys were explanted. A needle biopsy of the myocardium was obtained post mortem and histochemical and electron microscopy of cardiac muscle did not show findings consistent with NM [Figure 2].

## Discussion

We report a case of unexpected cardiac arrest and associated hypoxic-ischemic brain injury, in a child with typical congenital NM.

Due to early onset of symptoms, and the severity of muscle involvement being less than that seen in the severe congenital and intermediate congenital forms, typical congenital NM was diagnosed in this case.

NMs have been difficult to classify correctly through molecular testing, because of the size and heterogeneity of the genes involved. As a result, the prevalence of the various genetic causes of congenital NMs is largely unknown [14] and, genotype–phenotype correlation in NM remains largely unclear. In the aforementioned child, no mutation of ACTA1, TPM2 and TNNT1 genes was detected.

Several hypotheses have been proposed to explain sudden death in patients affected by NM, but the primary cause has not yet been clearly identified. Muscle weakness and gastroesophageal reflux have been described as cause of unexpected death in young patients with neuromuscular disorders. Few reports of sudden death in severe congenital (neonatal) form of NM have been described in infants under the age of 1 year, especially those under 6 months old [15] and are generally related to feeding. NM typically induces muscle weakness in proximal limb and truncal muscles, including respiratory and cardiac muscles. However, the degree of skeletal muscle weakness does not necessarily reflect the degree of respiratory muscle involvement, particularly in older children and adults [15]. In typical congenital NM, early mortality is not invariably due to hypotonia or severe weakness but rather respiratory insufficiency. Therefore, baseline and follow-up pulmonary function testing is recommended. In the patient here described, normal respiratory tests and nocturnal oximetry in the follow up period excluded a massive deterioration of pulmonary function due to NM.

Cardiac involvement is rare. The myocardium may be affected in the form of a dilated cardiomyopathy, while the involvement of the conduction system may result in arrhythmias and conduction defects, even in the absence of previous cardiomyopathy signs. The association between NM and hypertrophic or dilatative cardiomyopathy has been described in 6 children and 10 adults [4]. Electron microscopy studies of cardiac muscle were performed in 8 cases, but the typical rod-like structures were found in only 5 cases. In these patients, cardiac histological findings showed a few electron-dense fine structures related to Z lines and polymorphic materials (nemaline bodies), with multiple cytoplasmic nemaline rods in some fibers visible with Gomori trichrome staining [4]. Although cardiac involvement was considered as a result of ACTA1 mutations, the identification of NM cases with nonsense mutations, resulting in loss of giant sarcomeric protein function (extreme 5 C-terminal SH3 domain of nebulin), might explain the onset of conduction disorders, even in the absence of previous cardiomyopathy signs [2]. Giant sarcomeric proteins, modulating the calcium sensitivity of the contractile apparatus, protect the cardiac muscle against conduction defects. Thus, structural and/or histological and/or genetic abnormalities in giant sarcomeric proteins play a critical role in bearing high active stress and in fine-tuning the excitation-contraction coupling mechanism, leading to arrhythmias [2,4]. Moreover, mutations affecting actin polymerization (K336E), also leading to Ca2+ dysregulation, perturbation of muscle contractility, and, sequentially, to reduced force generation and transmission in cardiac muscle have also been reported [16].

In the reported case, echocardiography did not reveal signs of hypertrophic or dilatative cardiomyopathy. Electrocardiography showed a normal sinus rhythm. A post mortem cardiac biopsy was performed during organ explantation. The myocardic architecture was preserved and no changes consistent with the hypothesis of nemaline cardiopathy were observed under electron microscopy. In addition, ACTA1 gene mutation was not detected. Therefore, this case of sudden death differs from others. In children with severe neonatal NM, early unexpected death results from acute respiratory insufficiency or swallowing difficulties. A fatal course in adult and older children is only rarely due to cardiac failure, and cardiomyopathy is the most common cause. The cardiac arrest in the patient here described did not occur with respiratory failure or during feeding, and no signs of cardiomyopathy were detected. The child, despite the return of spontaneous circulation after cardiopulmonary resuscitation, died of massive hypoxic brain injury caused by prolonged cardiac arrest.

## Conclusions

Congenital typical NM is not necessarily a static or very slowly progressive disorder, and respiratory failure is the most common cause of death. Cardiac involvement is rare. We reported the case of a child with NM who died of cardiac arrest, described with a post mortem negative cardiac biopsy. Therefore, it is possible that acute cardiac deterioration can lead to early death in patients with NM.

## Consent

Written informed consent was obtained from the patient for publication of this Case report and any accompanying images. A copy of the written consent is available for review by the Editor-in-Chief of this journal.

## Abbreviations

RCP: Cardiopulmonary resuscitation
CT: Computed tomography
NEB: Nebulin
NM: Nemaline myopathy
ACTA1: Skeletal muscle α-actin
TPM2: Tropomyosin 2
TPM3: Tropomyosin 3
TNNT1: Troponin T1
